# CAPRIN1 (Cell Cycle-Associated Protein 1)-Related Neurodevelopmental Disorder: A Novel Mutation With Ataxia

**DOI:** 10.7759/cureus.103703

**Published:** 2026-02-16

**Authors:** Rebecca A Civan, Jessica Kottmeier, Richard Sidlow

**Affiliations:** 1 Faculty of Health Sciences, Ben-Gurion University of the Negev, Be'er Sheva, ISR; 2 Department of Pediatric Genetics, University of Missouri Healthcare, Columbia, USA

**Keywords:** autism, cerebellar-ataxia, developmental delay in childhood, neurodevelopmental disorder, stress granules

## Abstract

Mutations in the cell cycle-associated protein 1 (CAPRIN1) gene have been shown to present with language impairment, speech delay, intellectual disability, attention deficit hyperactivity disorder (ADHD), autism spectrum disorder (ASD), respiratory problems, limb and skeletal anomalies, developmental delay, feeding difficulties, seizures, ophthalmologic problems, cerebellar ataxia, dysmorphic features, and hearing loss. CAPRIN1 is involved in regulating the transport and translation of neuronal mRNAs, which encode for cell proliferation and migration proteins, and has been identified as a core component of stress granules. The majority of reported pathogenic mutations in the CAPRIN1 gene result in decreased protein levels and haploinsufficiency; however, they can also result in protein expansion.

We present the case of a patient with ASD, gross motor delay, fine motor delay, speech delay, mixed receptive-expressive language disorder, incontinence, and ADHD. Whole exome sequencing was significant for a likely pathogenic variant in the maternally inherited CAPRIN1 gene, c. 1045 C > T, p. (Q349*), with clinical correlation supporting a diagnosis of CAPRIN1-related neurodevelopmental disorder. Further analysis demonstrated that the patient’s likely pathogenic variant in the CAPRIN1 gene, c. 1045 C > T, p. (Q349*), was a nonsense mutation, de novo and heterozygous, likely resulting in loss of function of the CAPRIN1 protein. This novel mutation in the CAPRIN1 gene has not been previously described in the literature. This novel variant is consistent with the most common identified CAPRIN1 mutations, and his phenotypic presentation included the most common symptoms reported with CAPRIN1 mutations, including language impairment and speech delay, ADHD, ASD, respiratory symptoms, as well as ataxia.

## Introduction

Cell cycle-associated protein 1 (CAPRIN1) is an RNA-binding protein (RBP) that is encoded by the CAPRIN1 gene in humans [1]. CAPRIN1 encodes a ubiquitous protein that regulates the transport and translation of neuronal mRNAs critical for synapse malleability [1]. CAPRIN1 also encodes proteins involved in cell proliferation and migration in a variety of cell types [2]. CAPRIN1 is highly expressed in the central nervous system (CNS), especially in the cortex and cerebellum [3]. CAPRIN1 exhibits high expression in rapidly dividing cells and contributes to cell survival and proliferation in cancer cells [4]. As a result, CAPRIN1 is highly expressed in many malignant tumors [5]. Additionally, CAPRIN1 contributes to the viability and drug resistance of cancer cells through multiple mechanisms in the ubiquitination pathway [5]. In healthy tissues, CAPRIN1 localizes to the cytoplasm. However, CAPRIN1 is strongly expressed on the cell membrane surfaces of most solid cancers, but not in normal tissues [6]. These findings have identified CAPRIN1 as a potential target for cancer therapies [5-6].

CAPRIN1 has also been identified as a core nucleating component of stress granules [2,5,7]. Stress granules are non-membranous cytoplasmic structures that form under stress conditions within cells [7-8]. However, it has been shown that stress granules can be induced in the absence of functional CAPRIN1 protein [9]. Previous studies have shown that CAPRIN1-deficient mice exhibit early-onset progressive hearing loss, especially at mid to high frequencies, demonstrating that CAPRIN1 contributes to the maintenance of auditory function in the cochlea [9]. CAPRIN1 heterozygous knockout mice also exhibited reduced sociality, reduced preference for novelty, reduced flexibility, and difficulty in reversal learning, which are behaviors common in individuals with autism spectrum disorder (ASD) [10].

Case studies have demonstrated that mutations in CAPRIN1 may cause an ultra-rare neurodevelopmental disorder. Only 12 cases of CAPRIN1-related neurodevelopmental disorder have been previously described [3]. Most CAPRIN1 variants arise de novo, but inheritance from both affected and unaffected parents has been reported [3]. The majority of reported cases involve heterozygous loss-of-function mutations in the CAPRIN1 gene, resulting in decreased protein levels and haploinsufficiency [3]. However, in one identified case, a de novo heterozygous mutation of the CAPRIN1 protein resulted in protein expansion. In vitro, CAPRIN1 P521L aggregation was strongly enhanced, and the findings were consistent with a gain-of-function effect [3]. The CAPRIN1 gene contains at least 19 exons, and reported cases have identified CAPRIN1 mutations in exons 8, 9, 11, 14, and 16 through exon sequencing [3]. The majority of reported mutations were de novo; however, one mutation was inherited from an unaffected mother, and another was inherited from a mildly affected father [3].

Mutations in the CAPRIN1 gene can manifest with language impairment, speech delay, intellectual disability, attention deficit hyperactivity disorder (ADHD), ASD, respiratory problems, limb and skeletal anomalies, developmental delay, feeding difficulties, seizures, ophthalmologic problems, cerebellar ataxia, dysmorphic features, and hearing loss [3,11]. The phenotypic presentation of CAPRIN1 mutations varies significantly [11]. ASD and ADHD have been widely reported across multiple genetic variants; however, some patients have significant cognitive impairment, while others present with normal IQ [11]. Poor or absent language abilities have also been reported in the majority of known cases, with the presence of hearing loss, seizures, hyperkinetic movements, poor feeding and gastrointestinal issues, and dysmorphic facial features varying significantly among different reported CAPRIN1 mutations [3]. As CAPRIN1-related neurodevelopmental disorder is an extremely rare condition, phenotypic presentation can vary widely, and most known genetic mutations have only been reported in single individuals. Additional case reports describing newly identified individuals with CAPRIN1 mutations are critical for establishing genotype-phenotype correlations.

## Case presentation

Our patient was a five-year-old male with a history of ASD, gross motor delay, fine motor delay, speech delay, mixed receptive-expressive language disorder, incontinence, and ADHD. He was otherwise generally healthy and demonstrated appropriate growth patterns. His weight had consistently tracked along the 50th percentile curve, and his length had consistently tracked along the 85th percentile curve. He was normocephalic, with a head circumference within the 50th-98th percentiles. He was picky about his diet, but feeding difficulties had not been reported. Significant physical exam findings included a loose but productive cough with coarse upper airway sounds, as well as a wide-based gait with an ataxic quality to movement.

His parents reported behavioral concerns at school, including self-injurious behavior such as hitting himself, and the school expressed significant concerns regarding aggression, poor focus, self-isolation, and limited interaction with others. Parental reports on the ADHD rating scale were consistent with ADHD, combined presentation, based on symptoms of inattention and hyperactivity-impulsivity at levels meeting diagnostic criteria. The teacher-completed ADHD rating scale met criteria for ADHD, predominantly inattentive presentation. In the clinic, the patient was observed to transition rapidly from one item to another, with his entire body in motion even while seated. He received physical, occupational, and speech therapy through the school system and used an augmentative and alternative communication device. On the Peabody Developmental Motor Scales, Second Edition (PDMS-2, he scored at the 1st percentile in both grasping and visual-motor integration, placing him in the very poor range.

Activities of daily living, per parent report, included independently removing all clothing and demonstrating independence with donning pants and T-shirts; his mother assisted with adjusting his clothing. He was able to zip approximately 50% of zippers when they were threaded by his mother, but continued to have difficulty managing buttons. Regarding hygiene, he participated in bathing tasks, enjoyed brushing his teeth, and tolerated hair care without difficulty. For feeding, he preferred raw vegetables such as cucumbers and sweet peppers, as well as crunchy foods and fruits. He was able to use utensils but was noted to be significantly messy and frequently reverted to eating with his hands. He was in the process of toilet training, wearing diapers only at night, and voiding approximately once per hour during the day. He slept with his grandmother but fell asleep in his own bed.

On the Goldman-Fristoe Test of Articulation-3 (GFTA-3), the patient scored 28%, which is indicative of Apraxia. Errors observed include consistent final consonant deletion (FCD); fronting t/k and d/g; stopping b/f; t/ch, m/n, CR, occ WSD, and derhotacization. The patient demonstrated phonemic collapse to /d/ and the following phonemes: /g, k, b, sh, s, t, f, r, th, l, dg/. He demonstrated assimilation as well. Additionally, the patient was able to mark syllable shapes; however, he tended to mark them with /j/+vowel for the following phonemes: /m, s, g, n, v, sh, dg, r. Throughout, the patient demonstrated inconsistent errors and pronunciations and required models for most stimuli. Inconsistent vowel errors were also noted, including: ‘ah’/’ow’, ‘ee’/’uh’.

Clinical assessment of speech was conducted through chart review, parent report, and informal observation. Regarding receptive language skills, the patient followed one- to two-step directions for routine tasks and during play. He answered simple yes or no questions about basic attributes of common items and within play routines. He demonstrated understanding of simple WH-questions in context, such as “Where is the car?” and “What color?” He identified a variety of age-appropriate items during play. He had an expressive language device, but it was not currently being used. He was consistently producing increasingly intentional verbalizations consisting of one- to three-word approximations. His speech was generally intelligible within familiar contexts. The patient’s hearing was also evaluated. No concerns regarding hearing were reported, and there was no family history of childhood hearing loss. He passed the newborn hearing screen at birth. Otoscopy findings were clear bilaterally, with bilateral negative middle ear pressure noted. Hearing thresholds were found to be within the mild to normal range under headphones.

The patient had undergone a genetic evaluation at five years old due to hypotonia, developmental delay, autism, ataxic gait, and dysmorphic features. Notable head and neck findings on physical exam included metopic prominence, tapering of the lateral third of eyebrows, mild downward slanting of palpebral fissures, wide nasal bridge, depressed and upturned nasal tip, hypoplastic malar configuration, small dysplastic ears, and long upper lip with hair. Head shape, face shape, hair, mouth, gums, palate, chin, and neck were normal. Additional abnormalities included hirsute back, left lower extremity longer than right lower extremity, and third toes incurving under second toes bilaterally. Upper extremities, skin, and genitalia were found to be normal. On neurologic exam, the patient demonstrated mild global hypotonia.

Trio whole-exome sequencing analysis identified a heterozygous, maternally inherited, likely pathogenic variant in CAPRIN1, c. 1045 C > T/p. Q349* (Genedx, Gaithersburg, MD). The mitochondrial genome was evaluated and was negative, and no secondary findings were identified.

## Discussion

We describe the case of a five-year-old boy with a novel mutation in the CAPRIN1 gene, which has not been described in the literature to date. CAPRIN1 is involved in regulating transport and translation of neuronal mRNAs for synapse malleability, encoding for cell proliferation and migration proteins in a variety of cell types, exhibits high expression in rapidly dividing cells, and contributes to cell survival and proliferation in cancer cells, and has been identified as a core component of stress granules [1,2,8]. Reported mutations in the CAPRIN1 gene have been shown to present with language impairment, speech delay, intellectual disability, ADHD, ASD, respiratory problems, limb and skeletal anomalies, developmental delay, feeding difficulties, seizures, ophthalmologic problems, cerebellar ataxia, dysmorphic features, and hearing loss [3,11]. Clinical features of all known patients with CAPRIN1 neurodevelopmental disorder, including this patient, are summarized in Table 1 (adapted from Pavinato et al.) [11].

**Table 1:** Clinical features of CAPRIN1 patients Adapted from Pavinato et al. [11], with the addition of the present case (Case 13). (+): reported in the patient; for ID/LD: +: mild, ++: moderate, +++: severe; (-): Not reported in the patient; NA: information not available; F: female; M: male. ADHD: attention deficit hyperactivity disorder; ID: intellectual disability; LD: learning disability

Case	1	2	3	4	5	6	7	8	9	10	11	12	13
Sex	M	M	M	F	M	F	M	M	M	M	M	F	5
Age at examination, years	7	12	6	17	10	8	7	7	12	48	3	10	M
Language impairment	+	+	+	+	+	+	+	+	+	+	+	+	+
ASD	+	+	+	+	+	-	+	+	-	-	+	-	+
ID/LD	-	++	+	+	++	-	+++	+	++	+	+	+++	++
ADHD	+	+	-	+	+	+	NA	+	+	+	-	+	+
Developmental delay	-	+	+	-	+/-	-	-	-	+	-	+	-	+
Other behavioral features	+	-	-	+	-	+	-	-	+	+	+	+	+
Seizures	-	+	-	+	-	-	-	-	-	-	+	+	-
EEG anomalies	NA	+	-	-	-	NA	NA	NA	NA	NA	+	+	NA
Hands and feet malformations	-	+	+	+	+	-	-	-	+	-	-	+	+
Breathing problems	-	-	-	+	+	+	-	+	+	+	-	-	+
Ocular problems	-	-	-	+	+	-	-	-	+	+	-	-	-
Hearing problems	-	-	+	+	-	-	-	-	-	+	-	-	-

The patient presented with ASD, gross motor delay, fine motor delay, speech delay, mixed receptive-expressive language disorder, incontinence, and ADHD. Whole exome sequencing was significant for a likely pathogenic variant in the maternally inherited CAPRIN1 gene, c. 1045 C > T, p. (Q349*), with clinical correlation suggested to support a diagnosis of CAPRIN1-related neurodevelopmental disorder. The variant was classified as likely pathogenic based on American College of Medical Genetics and Genomics and the Association for Molecular Pathology (ACMG/AMP) criteria, including PVS1 (predicted loss-of-function variant in a gene in which loss of function is a known disease mechanism) and PM2 (extremely low frequency in the Genome Aggregation Database [gnomAD]). The patient’s developmental delay, language impairment, ASD, ADHD, ataxia, and respiratory findings on physical exam were consistent with known features and presentation of CAPRIN1-related neurodevelopmental disorder [3,11]. The likely pathogenic variant identified in this patient occurred at residue 349, immediately adjacent to residues 352-380 [8].

The novel CAPRIN1 genetic mutation found in this patient was maternally inherited and heterozygous. This is consistent with known CAPRIN1 mutations [3]. Of the 12 other reported patients with CAPRIN1 mutations, all were heterozygous, and two additional known patients had inherited mutations. Additionally, 10 of the previously reported mutations resulted in loss-of-function mutations, while two patients with cerebellar ataxia and cognitive decline were found to have mutations with a gain-of-function effect [3,11]. The variant in this patient is presumed to cause loss of function of CAPRIN1 and result in protein truncation or nonsense-mediated decay, but this has not been confirmed. RNA transcriptomics were ordered to verify the pathogenicity of the patient’s truncating variant. However, this could not be obtained due to insurance limitations. Out of the 10 loss-of-function mutations reported, six were nonsense mutations, two were splice site mutations, and two were frameshift mutations [3,11].

**Figure 1:**
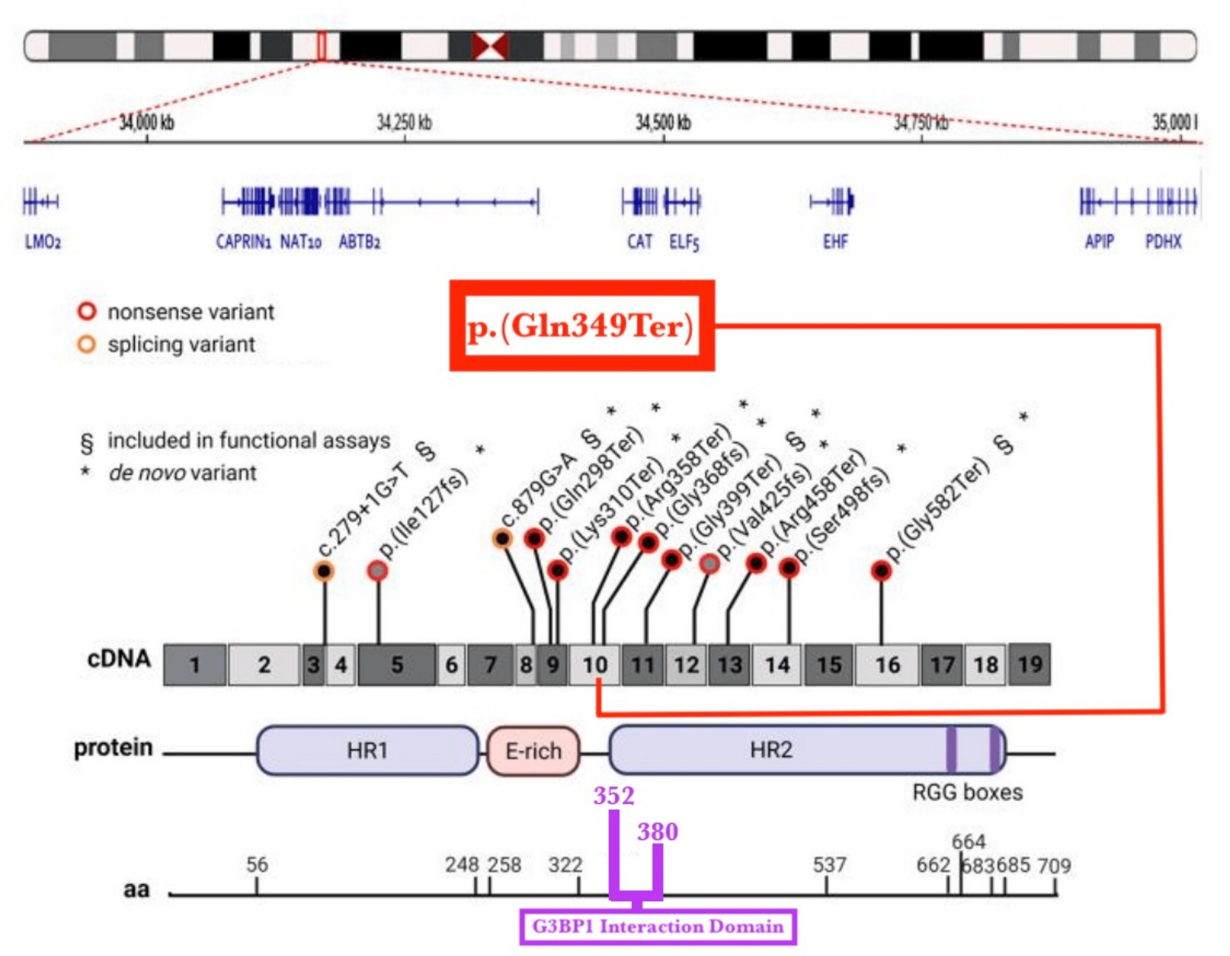
CAPRIN1 neurodevelopmental disorder genetic variants Adapted from Pavinato et al. [11]. Schematic representation of a large ∼1.4 Mb deletion spanning the CAPRIN1 gene (top) and 11 CAPRIN1 (NM_005898.5) loss-of-function variants (bottom); 10 single-nucleotide variants reported in Pavinato et al. [11], and two variants reported in the literature are represented on a schematic view of the protein (created with BioRender.com). The CAPRIN1 presumed loss-of-function, likely pathogenic variant described in this report (NM_005898.4) was added to the figure, demonstrated in the red box, p. (Gln349Ter). The G3BP1 interaction domain, composed of the 29 amino acid sequence 352-380, is highlighted in purple (bottom of figure)

The site of presumed truncation in this patient was located at residue 349, immediately adjacent to residues 352 to 380 [8]. Previous studies have demonstrated that CAPRIN1 interacts with Ras-GTPase-activating protein binding protein 1 (G3BP1), nuclear transport factor 2-like (NTF2L) at this domain [8]. The 29-amino acid sequence 352-380 has previously been identified as sufficient for recognition by G3BP-1 [8]. This is significant because the CAPRIN1/G3BP-1 complex likely regulates the transport and translation of mRNA-encoding proteins involved in synaptic plasticity in neurons and in cellular proliferation and migration in multiple cell types [12]. This domain also includes Gly368 and Phe372, which are critical for the interaction between G3PB1 and CAPRIN1, which mediates the reversible assembly of stress granules [8]. The site of presumed truncation in this patient, its location relative to other known CAPRIN1 pathogenic variants, and its location relative to the G3BP1 interaction domain are demonstrated in Figure 1 (adapted from Pavinato et al.) [11].

Of the 12 previously reported cases, the neurodevelopmental phenotype consisted of language impairment or speech delay in all, intellectual disability in 83%, ADHD in 82%, and ASD in 67% of cases [11]. Language impairment and speech delay, ADHD, and ASD were all present in this patient. Additionally, of the 12 previously described patients, 50% had respiratory problems, 50% had limb or skeletal anomalies, 42% had developmental delay, 33% had feeding difficulties, 33% had seizures, and 33% had ophthalmologic problems [11]. This patient did not have a respiratory diagnosis, but he presented with a loose, productive cough and coarse upper airway sounds on physical examination, possibly indicating an undiagnosed respiratory condition related to his mutation. He was reported to be a picky eater, a behavior often associated with ASD, but he was not reported to have feeding difficulties and was maintaining consistent weight gain and height velocity, according to his growth curves. The patient was not found to present with seizures, limb or skeletal anomalies, or vision problems. The two patients with the reported missense mutations resulting in gain-of-function effects presented with ataxia, which was also present on examination in this patient [3].

## Conclusions

We discussed a case of a five-year-old male presenting with ASD, gross/fine motor delay, speech delay, mixed receptive-expressive language disorder, incontinence, ataxia, and ADHD, who exhibited a novel CAPRIN1 mutation. The patient’s novel likely pathogenic variant in the maternally inherited CAPRIN1 gene, c. 1045 C > T, p. (Q349*), was found to be a nonsense mutation, heterozygous, likely causing loss of function of the CAPRIN1 protein, consistent with the most commonly identified CAPRIN1 mutations. He also manifested many of the most common symptoms reported with CAPRIN1 mutations, language impairment and speech delay, ADHD, ASD, respiratory symptoms, as well as ataxia. As CAPRIN1 neurodevelopmental disorder is an extremely rare condition, documentation of this previously unreported variant further supports emerging genotype-phenotype correlations for this disorder.
